# Diagnosis of Citrus Greening Using Artificial Intelligence: A Faster Region-Based Convolutional Neural Network Approach with Convolution Block Attention Module-Integrated VGGNet and ResNet Models

**DOI:** 10.3390/plants13121631

**Published:** 2024-06-13

**Authors:** Ruihao Dong, Aya Shiraiwa, Achara Pawasut, Kesaraporn Sreechun, Takefumi Hayashi

**Affiliations:** 1 Faculty of Informatics, Kansai University, Takatsuki 569-1095, Osaka, Japan; k975945@kansai-u.ac.jp; 2 Electrical Engineering and Computer Science, Tottori University, Tottori 680-8552, Tottori, Japan; shiraiwa@tottori-u.ac.jp; 3 Royal Project Foundation, 910 Moo 3, T. Maehia, Muang, Chiang Mai 50200, Thailand; acrpwst@gmail.com (A.P.); hrhki2015@gmail.com (K.S.)

**Keywords:** attention mechanism, CNN models, deep learning, object detection, plant disease detection, transfer learning

## Abstract

The vector-transmitted Citrus Greening (CG) disease, also called Huanglongbing, is one of the most destructive diseases of citrus. Since no measures for directly controlling this disease are available at present, current disease management integrates several measures, such as vector control, the use of disease-free trees, the removal of diseased trees, etc. The most essential issue in integrated management is how CG-infected trees can be detected efficiently. For CG detection, digital image analyses using deep learning algorithms have attracted much interest from both researchers and growers. Models using transfer learning with the Faster R-CNN architecture were constructed and compared with two pre-trained Convolutional Neural Network (CNN) models, VGGNet and ResNet. Their efficiency was examined by integrating their feature extraction capabilities into the Convolution Block Attention Module (CBAM) to create VGGNet+CBAM and ResNet+CBAM variants. ResNet models performed best. Moreover, the integration of CBAM notably improved CG disease detection precision and the overall performance of the models. Efficient models with transfer learning using Faster R-CNN were loaded on web applications to facilitate access for real-time diagnosis by farmers via the deployment of in-field images. The practical ability of the applications to detect CG disease is discussed.

## 1. Introduction

Citrus Greening (CG) disease, caused by the pathogen *Candidatus* Liberibacter asiaticus, is a destructive disease of citrus that is spread by grafting or transmitted by the vector insect citrus psyllid [1]. The most typical symptoms of the disease are “blotchy mottling”, partial yellowing of green leaves, leaf-thickening, and corking of veins [2]. As the disease develops, infected trees gradually decline and finally die. No curable measures for this disease have been developed for practical citrus cultivation. The current management of CG thus mainly involves insecticide application to control vectors and frequent surveillance of trees to detect and remove infected trees as soon as possible. Hence, the detection of diseased trees is one of the most important management practices, as it can reduce the risks of both primary and secondary infections. The most widely adopted disease-detection practice is the use of Polymerase Chain Reaction (PCR) [3], which requires collecting plant materials from trees and subsequently processing them for chemical analysis to detect pathogen genes in the samples. However, this requires skills in chemical experiments, is time- and labor-intensive, and requires a long time to obtain results. These conditions make growers reluctant to use the method. Therefore, there is a demand for the development of simple and rapid diagnostic methods.

Digital image analyses for plant disease diagnosis are increasingly being used. Convolutional neural networks (CNNs) incorporating image analyses of individual plant leaves have been examined as classification models, particularly under a controlled laboratory environment [4]. For instance, deep learning techniques with machine learning are used in citrus disease diagnosis systems [5]. This approach, applied to a dataset with five categories of leaves based on disease development, attained an average accuracy of 87% on test sets. Prior to the application, five categories of healthy and diseased leaf images of citrus should be defined to implement transfer learning with VGG19 and AlexNet models, which successfully distinguish the groups with 94.3% average accuracy on the test set [6]. Although the approach can perform well in a laboratory under stable conditions, its reliability is limited in fields where conditions, e.g., weather, light conditions, and background noise, easily vary [7]. The other factor that seriously affects the accuracy of image analyses is the coexistence of other diseases with symptoms similar to CG, easily reducing the confidence of the analyses.

On the other hand, object-detection technology using deep learning techniques is used to identify and locate specific objects (targets) in images and videos. This procedure is widely applied in the diagnosis of plant diseases. Although this technique is computationally expensive, it can recognize different categories of objects and draw bounding boxes around each of them. An optimized YOLO-V4 model was used to examine six different disease images obtained from fruits in a citrus orchard [8]. The EfficientNet model was used for classification and achieved 84.2% accuracy in CG disease. This model was implemented with different object detection models to effectively detect citrus disease by focusing on the spot where the symptoms occur [9]. As citrus psyllid is the only insect vector of CG disease, Dai F et al. [10] aimed to prevent CG disease by detecting citrus psyllids on citrus leaves taken from a natural environment and achieved an average precision of 90.21%.

The above approaches have been tested for their ability to detect various targets, but their availability remains to be studied. The diagnosis can be made when the trees bear fruits, but this is not practical for early diagnosis and management [8]. The research in [9] attempted to identify diseases from the fine features of citrus leaves, but since symptoms of CG disease appear across the entire leaf, it was difficult to make judgments based on specific localized areas; thus, they did not consider diagnosing CG disease. Another study [10] has the potential to help prevent the early spread of CG disease, but because the target is too small, it is difficult to grasp the overall situation in an orchard. Additionally, it cannot be used for detecting images of CG disease that do not contain vector insects.

This study reports on how a simple and precise diagnostic system for CG disease can be developed, focusing on the following issues:

1. A non-invasive method that involves collecting high-resolution, in-field images taken in the natural environment of an orchard and performing annotations of leaves on branches;
2. A diagnostic approach using the Faster R-CNN object detection architecture, enabling simultaneous identification and localization of CG disease, thereby improving detection efficiency;
3. The integration of the Convolution Block Attention Module (CBAM) attention mechanism into the VGGNet and ResNet models to improve CG disease detection capability;
4. The development of a web application tool for real agricultural scenarios.

This system was examined to determine whether it could quickly determine the CG-infection status of leaves by simply photographing citrus branches without the interaction by the location or background noises. Based on the results, this study considered the potential of the tested models for practical uses by growers.

## 2. Results

### 2.1. Results of VGGNet and ResNet

The effectiveness in detecting CG disease by the 5-fold CV using the VGGNet and ResNet models is ranked as follows: VGG19 < VGG16 < ResNet50 < ResNet152 < ResNet101, with ResNet101 recording the highest AP of 85.07% (Table 1). ResNet50 distinguished the two categories “healthy” and “others” most effectively, achieving an AP of 91.29%, leading to the highest overall model performance with an mAP. Additionally, the AP for all categories was higher for ResNet models than for VGGNet models, indicating that ResNet models performed more stably and comprehensively.

**Figure 1:**
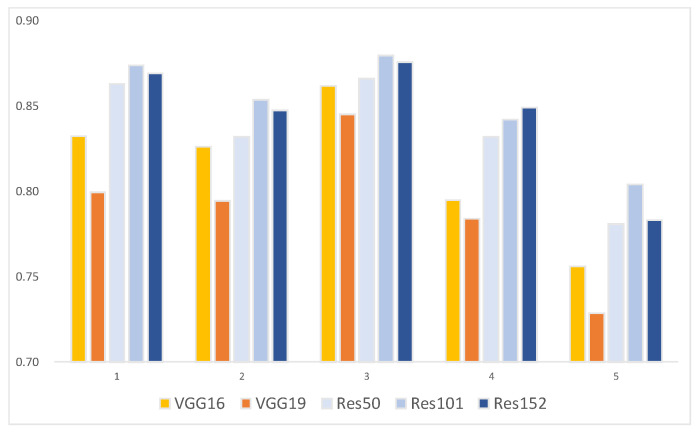
AP of “greening” with VGGNet and ResNet in each experiment.

**Figure 2:**
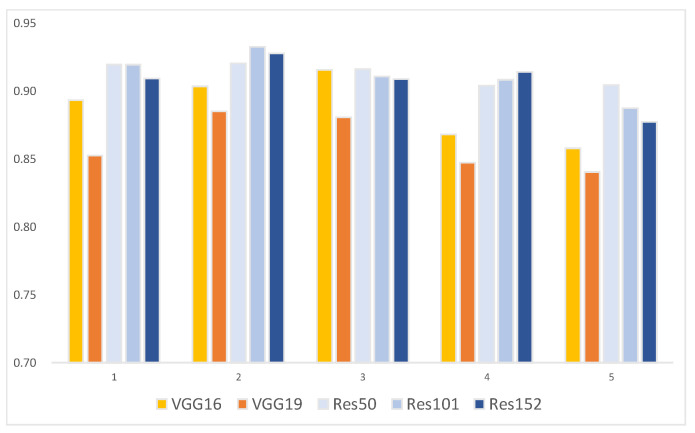
mAP of VGGNet and ResNet in each experiment.

The AP with the 5-fold CV distinguished the “greening” category (Table 2 and Figure 1) best by ResNet101 in the experiment (3/5), achieving an AP of 87.96%. The mAP (Table 3 and Figure 2) for all categories was highest with ResNet101 in the experiment (2/5), reaching 93.25%. Moreover, except for experiment (3/5), where VGG16’s mAP outperformed both ResNet101 and ResNet152, ResNet models generally outperformed VGGNet in both CG disease identification ability and overall model performance.

### 2.2. Results of VGGNet+CBAM and ResNet+CBAM

Based on the 5-fold CV with CBAM, the performance in CG disease detection was ranked as follows: VGG19+CBAM < VGG16+CBAM < ResNet50+CBAM < ResNet101+CBAM < ResNet152+CBAM (Table 4), with the highest AP of 86.52% achieved by ResNet152+CBAM. ResNet101+CBAM showed the best performance for the “healthy” category, while ResNet152+CBAM performed best for the “others” category. Overall model performance was slightly better in ResNet152+CBAM than in ResNet101+CBAM, providing the highest mAP of 92.33%.

**Figure 3:**
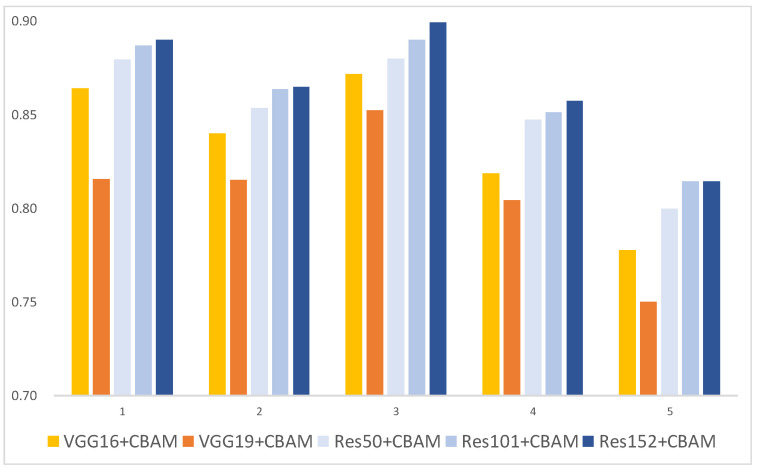
AP of “greening” with VGGNet+CBAM and ResNet+CBAM in each experiment.

**Figure 4:**
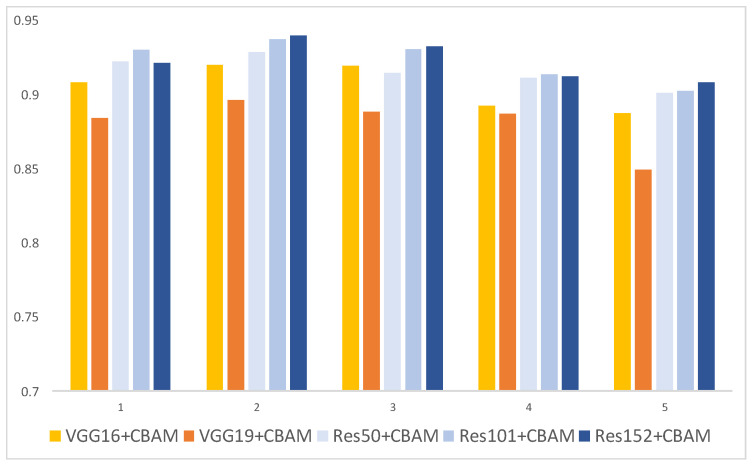
mAP of VGGNet+CBAM and ResNet+CBAM in each experiment.

The AP (Table 5 and Figure 3) with a 5-fold CV distinguished the “greening” category best in the experiment (3/5) with ResNet152+CBAM, achieving an AP of 89.92%. The mAP (Table 6 and Figure 4) for all categories was highest in the experiment (2/5) with ResNet152+CBAM, reaching 94.02%. Additionally, except for experiment (3/5), where VGG16+CBAM’s mAP surpassed ResNet50+CBAM, ResNet+CBAM models generally outperformed VGGNet+CBAM models in both CG disease distinction and overall performance across all categories.

### 2.3. Comparison with the Integration of CBAM

**Figure 5:**
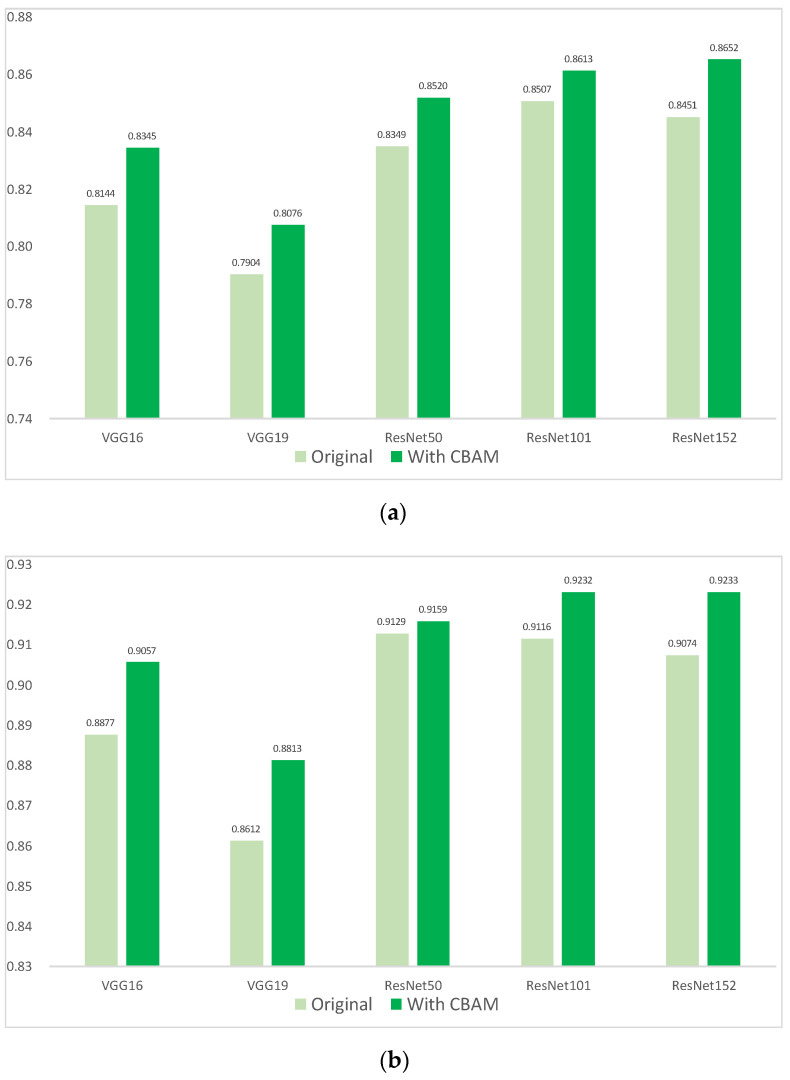
Improvement in each model by integrating CBAM. (**a**) AP of “greening”; (**b**) mAP.

The integration of CBAM increased both the performance distinction of CG disease and the overall performance of all models (Figure 5). Before the integration of CBAM, ResNet101 achieved the highest AP of 85.07% for the “greening” category, while ResNet50 attained the highest mAP of 91.29%. After integrating CBAM, notably, the ResNet152+CBAM model showed an enhancement in CG disease detection by 1.45%, reaching the highest AP of 86.52%. Concurrently, the overall model performance exhibited an improvement of 1.04%, achieving the highest mAP of 92.33%.

### 2.4. ResNet152+CBAM

**Figure 6:**
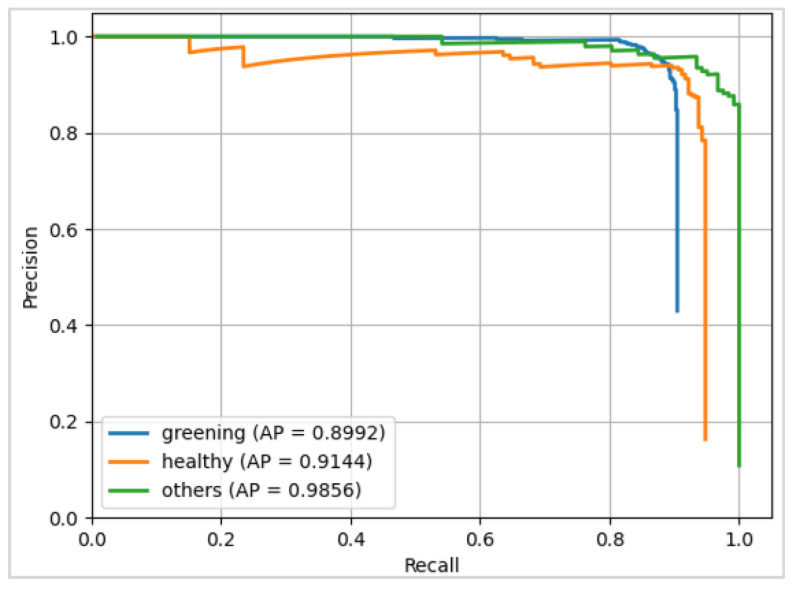
The precision–recall curve of ResNet152+CBAM in experiment (3/5).

The ResNet152+CBAM model achieved the highest AP (89.92%) for CG disease in the experiment (3/5) (Table 5). The precision–recall curve of this model (Figure 6) shows the precision (y-axis) against the recall (x-axis) for different probability thresholds, and the area under each curve presents the AP of each category. Although the AP for CG disease was slightly less than 90%, it reached 91% for the healthy category and more than 98% for other diseases.

**Figure 7:**
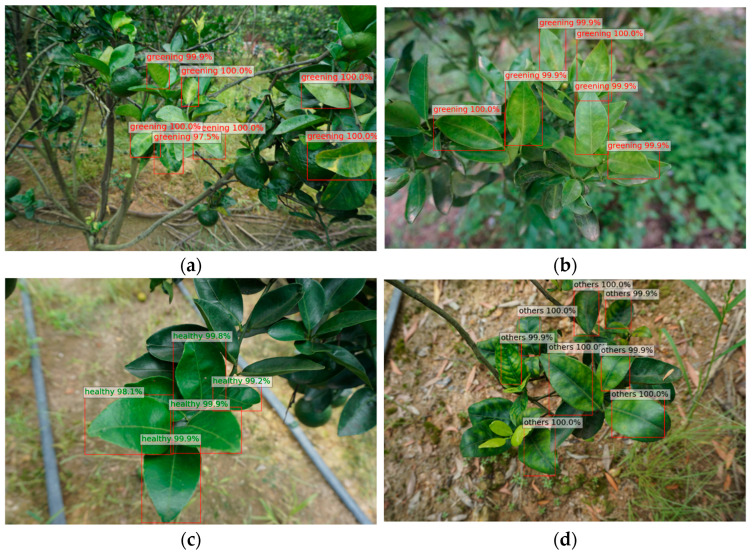
The detection results based on ResNet152+CBAM in the experiment (3/5). (**a**,**b**) Samples of CG-affected leaves; (**c**) A sample of healthy leaves; (**d**) A sample of leaves with other diseases.

The availability of the ResNet152+CBAM model as a CG disease diagnosis tool for agricultural practices was evaluated using the web application introduced in Section 4.6 of this paper. The images used were new in this evaluation, and the probability threshold was set to 0.8, meaning that an instance was only classified as positive if the model predicted it with more than 80% confidence. The model correctly detected CG-symptomatic leaves with multiple objects, even at positions slightly away from citrus leaves (Figure 7).

**Figure 8:**
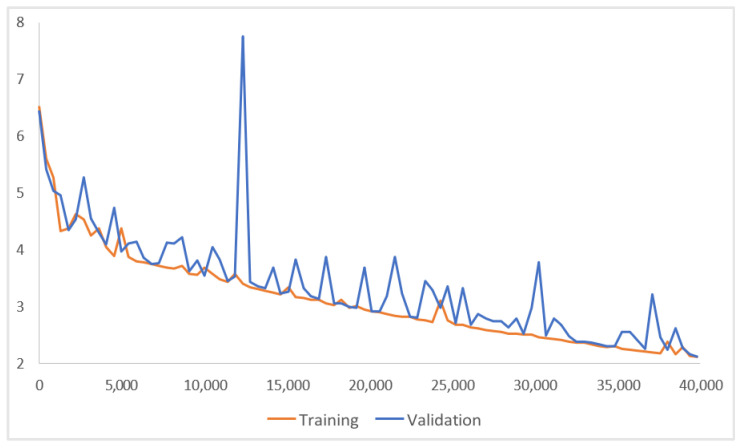
The total loss curve of ResNet152+CBAM in the experiment (3/5).

The total loss curves of both the training and validation sets of this model (Figure 8) were used to examine whether the model exhibits overfitting or underfitting during the training process. The total loss, which corresponds to the sum of two classification losses (identifying what those objects are), two regression losses (determining where the objects are), and a regularization loss (to prevent overfitting), helped prevent the models from overfitting and contributed to fitting the distribution of the new data. This means that as more training iterations were conducted, the total loss on the training set steadily decreased. Although the validation set loss fluctuated, it tended to decrease as well. Therefore, the model progressively learned and fit the features of the training data in the right direction.

## 3. Materials

### 3.1. Dataset

**Figure 9:**
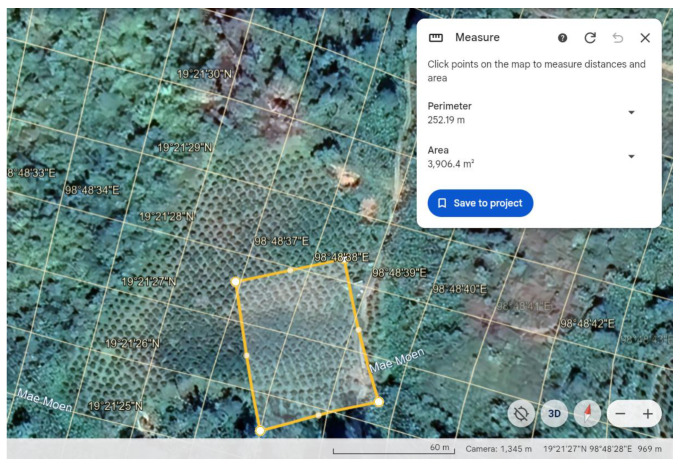
Experimented field of the orchard in Thailand from Google Earth.

The experimental field in this study covered approximately 4000 m^2^ and contained around 120 trees of the local mandarin variety Sai Num Phung, *Citrus reticulata*. The orchard was located in Mae Na, Chiang Dao District, Chiang Mai, Thailand (Figure 9, 19°21′26″ N 98°48′38″ E, 969 MSL). Trees were planted with 3 m spacing between rows and 4 m spacing between trees within individual rows. The ages of the trees ranged between 5 and 7 years. A total of 20 leaves per tree were randomly collected from 60 trees, and CG infection was confirmed with a Polymerase Chain Reaction (PCR) test conducted by Highland Research and Development Institute (HRDI), which revealed 24 CG-infected and 36 non-infected trees. Branches from these 60 trees, each with at least 5 extended leaves, were photographed from approximately 40 cm away on a sunny afternoon between 13:00 and 17:00 on 12 January 2021. A total of 82 images capturing leaves were used in this study. All images were obtained with a digital camera (ILCE-6000, Sony, Tokyo) at a resolution of 6000 × 4000 pixels.

### 3.2. Data Augmentation

**Figure 10:**
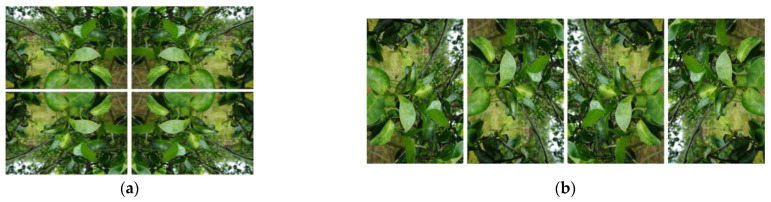
Data augmentation with flipping horizontally (**a**) or vertically (**b**) at 90 ° rotation.

Data augmentation is a method of artificially increasing the size of an existing training dataset to improve the performance of deep learning models [11]. This process is commonly used to enhance the model’s generalization ability and improve performance on new, unseen data. Various data-augmentation techniques, such as image rotation, flipping, scaling, cropping, color adjustment, and noise addition, have been instrumental in preventing overfitting and contributing to model performance improvement, especially in cases of small datasets [12]. In this study, the 82 images obtained were flipped horizontally/vertically or rotated by 90 degrees, resulting in a total of 656 images (Figure 10).

### 3.3. Annotation

**Figure 11:**
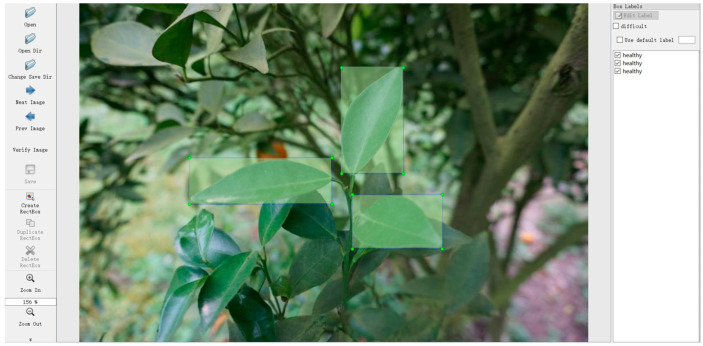
A sample of annotation. Bounding boxes were drawn around the leaves, and the results of expert visual assessments were attached.

**Figure 12:**
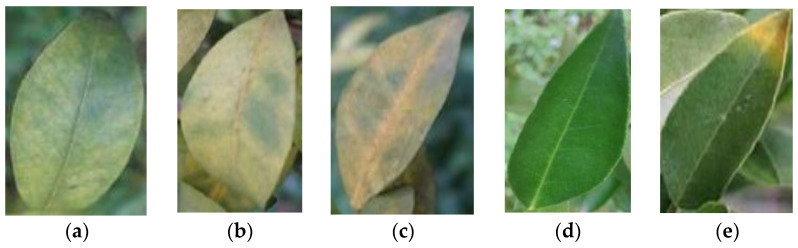
Samples of leaves with different symptoms in annotations. (**a**–**c**) CG-infected leaves with different symptoms were annotated as “greening”; (**d**) non-symptomatic healthy leaves were annotated as “healthy”; (**e**) leaves with other disease symptoms were annotated as “others”.

Deep learning with annotation, where correct labels are manually attached to data such as images, text, and audio, helps the model understand what it should learn. The quality of annotations directly affects the model’s accuracy when it processes new data, making it a crucial element for the success of supervised learning tasks [13]. In the present study, the open-source tool “LabelImg [14]” was adopted to create annotations. This tool is used to identify and define bounding boxes for ground truth positions within the images. Leaves in each image were annotated, as shown in Figure 11, where the leaves to be analyzed were depicted with squares. The annotation process was based on the results of visual inspections by experts with more than 20 years of experience to ensure accuracy. To maximize the robustness of the classification, we defined the following three categories for the model application: “greening” for symptomatic CG-infected leaves, “healthy” for non-symptomatic healthy leaves, and “others” for leaves showing symptoms of other diseases, as shown in Figure 12. The numbers of annotated leaves in each of the three categories are shown in Table 7.

## 4. Methods

### 4.1. Faster R-CNN-Based Diagnosis System

**Figure 13:**
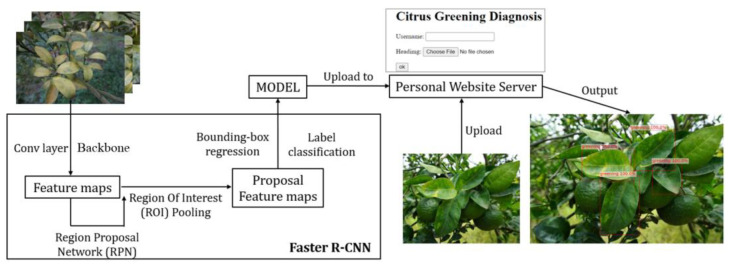
The schematics of the Faster R-CNN based Diagnosis System.

The Faster R-CNN [15] is a type of deep learning architecture designed to locate and identify objects within multi-scale images, achieving high accuracy in object-detection tasks. Its capability to fine-tune parameters for specific datasets makes it particularly effective for transfer-learning applications. Figure 13 outlines our diagnosis system’s configuration, which utilizes the Faster R-CNN framework. In this system, the input images are resized to 900 × 600 pixels and processed through the Faster R-CNN for training, resulting in a well-trained model. This model is hosted on a web server, facilitating an online CG disease diagnosis platform. Users can upload images of any size via a web application and receive results featuring bounding boxes, labels, and confidence scores of the identified targets.

### 4.2. Backbone and Transfer Learning

The backbone is a CNN model located in the initial stages of object-detection models like Faster R-CNN, playing a crucial role in extracting useful features from the input image. By using a backbone, it is possible to capture features ranging from low-level characteristics such as edges, textures, and shapes to more advanced abstract features. Therefore, the method of using a pre-trained CNN model as a backbone through transfer learning is widely employed to adapt to new tasks [16].

Transfer learning involves applying the knowledge (weights and feature extractors) of a model trained on a large-scale task as initial values for another specific task. This can reduce the amount of data required for learning, shorten the learning time, and improve performance, especially when the data are scarce or the task is complex [17]. When using transfer learning with Faster R-CNN, its capability to capture various image features allows it to immediately provide high-level feature-extraction abilities for new object-detection tasks [15].

In this study, we utilized pre-trained models based on the ImageNet subset of the ImageNet Large Scale Visual Recognition Challenge (ILSVRC) [18] as the backbone of Faster R-CNN. The ILSVRC is a large-scale database composed of over 1.2 million annotated images, encompassing more than 1000 object categories, and is widely used as a standard benchmark in many computer vision studies. Specifically, the pre-trained models used in this study were VGGNet and ResNet, which have demonstrated high performance across various tasks.

#### 4.2.1. VGGNet

VGGNet [19], a profound CNN model, is highly regarded in the field of image classification for its simplicity and uniform design. It is characterized by multiple convolutional layers with small 3 × 3 filters, each followed by max-pooling layers. The defining feature of VGGNet is its repetitive stacking of convolutional layers, enabling deeper representations [19]. VGGNet has several variations, with VGG16 and VGG19 being the most used. VGG16 consists of 13 convolutional layers and 3 fully connected layers, while VGG19 has an additional 3 convolutional layers, enhancing its ability to capture more complex features. In this study, we utilized these two pre-trained VGGNet models for transfer learning.

**Figure 14:**
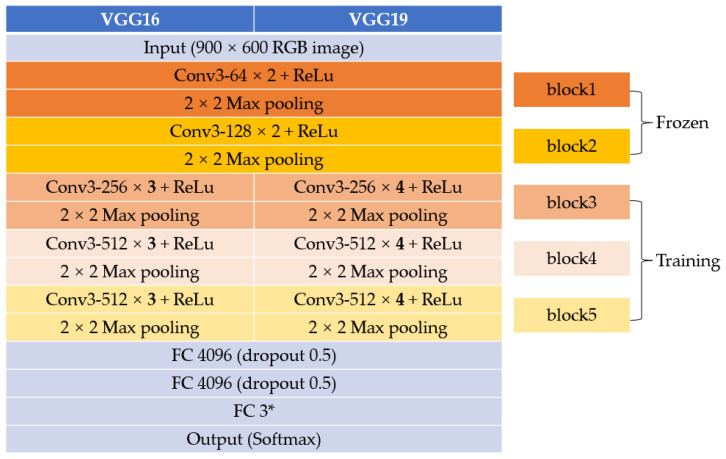
The structure of VGGNet (VGG16, VGG19). * A three-class classification task.

In transfer learning, a common approach involves freezing certain blocks of the network. This means maintaining the weights of these frozen blocks as unchanged while updating the weights of the other layers during training. This strategy effectively leverages pre-trained knowledge while tailoring the model for a new task, especially with limited data [20]. In our study, we found that freezing the first two blocks of VGGNet yielded optimal training performance. The architecture of our employed VGGNet is illustrated in Figure 14.

#### 4.2.2. ResNet

ResNet [21] is an innovative deep learning model that can effectively train deep networks and is considered the benchmark model for most computer vision tasks. Previous CNN models before ResNet tended to suffer from vanishing or exploding gradients as the network deepened, making learning difficult [20]. ResNet introduced the “residual block” structure, using “skip connections” that add the input directly to the output, effectively avoiding these issues.

**Figure 15:**
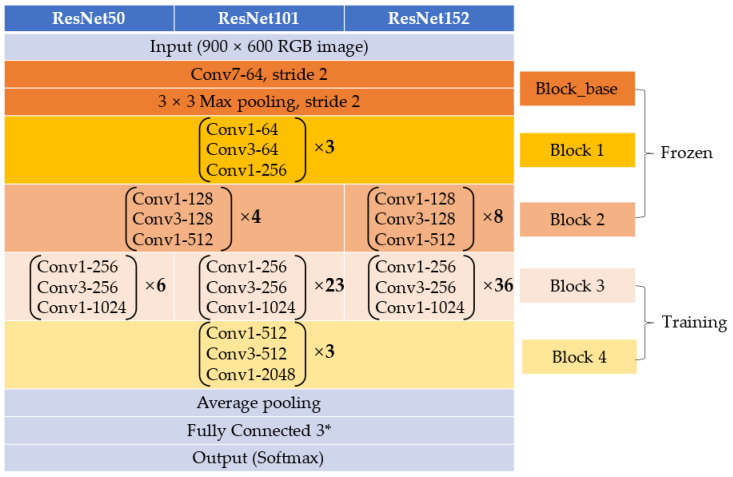
The structure of ResNet (ResNet50, ResNet101, and ResNet152). * A three-class classification task.

There are various ResNet model variations. In this study, we adopted the ResNet50, ResNet101, and ResNet152 models, which have demonstrated effectiveness across a wide range of computer vision tasks. To achieve optimal learning effects in transfer learning, experiments were conducted similarly to VGGNet, and it was found that freezing the layers before the third block was optimal. The structure of the ResNet models used is shown in Figure 15.

### 4.3. Attention Mechanism

When reading a text, one may “pay attention” to certain words according to the context and deeply understand their meaning. The attention mechanism in deep learning models attempts to mimic this human process. Since the advent of the Transformer [22] model, the attention mechanism has garnered significant interest, especially in natural language processing, and has since been widely applied to other areas like image recognition. By using the attention mechanism, deep learning models can focus on important parts of the data, making it a powerful tool that improves task performance [23]. In this study, to achieve higher diagnostic performance, we integrated the CBAM attention mechanism into the two types of backbone models mentioned in Section 3.2 and conducted experiments.

#### 4.3.1. CBAM

**Figure 16:**
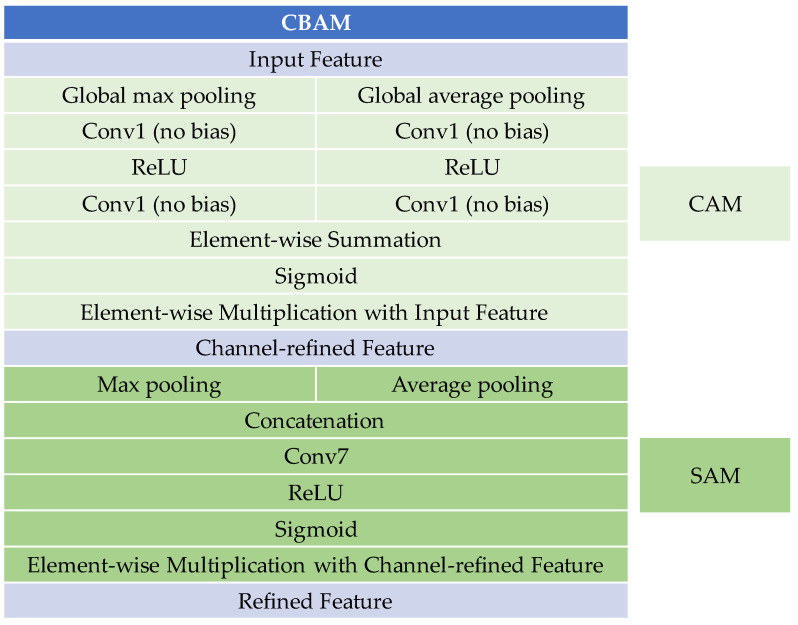
The structure of the CBAM attention mechanism.

The CBAM [24] is designed to bolster the representational capabilities of CNNs by focusing on both spatial and channel-wise attention. CBAM comprises two sub-modules: the Channel Attention Module (CAM) and the Spatial Attention Module (SAM). Figure 16 shows the structure of the CBAM.

CAM concentrates on the inter-channel relationships of features, emphasizing "what" aspects of input images are meaningful. The process starts with global max pooling and global average pooling of the input features, each then fed into a two-layer neural network. The reduction ratio parameter adjusts the neuron count reduction in the first layer, followed by ReLU activation. The second layer’s neuron count is restored to its original number. The outputs are then combined using element-wise summation and passed through a sigmoid activation, leading to a channel-refined feature for SAM.

SAM leverages the inter-spatial relationships of features, focusing on "where" informative parts are located. The channel-refined feature undergoes channel-based max pooling and average pooling, followed by concatenation. A 7 × 7 convolution operation with ReLU and sigmoid activation is then applied. Finally, an element-wise multiplication with the channel-refined feature generates the refined features.

#### 4.3.2. Proposed Model

**Figure 17:**
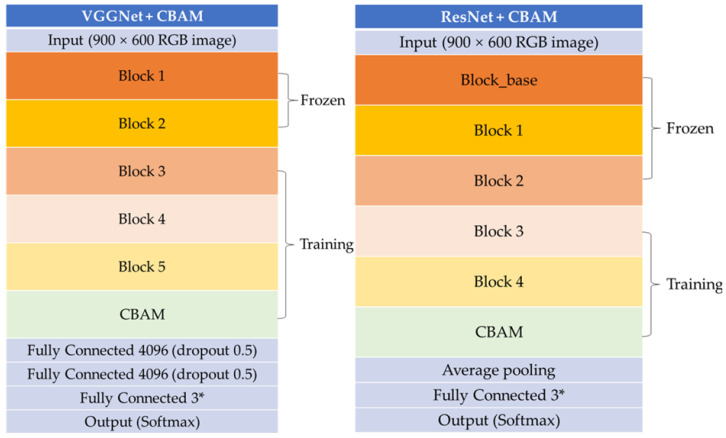
The structure of our proposed model (VGGNet + CBAM and ResNet + CBAM). * A three-class classification task.

CBAM is a lightweight module that can be seamlessly integrated into any position within any CNN model [24]. Chougui A. et al. [25] demonstrated enhanced feature extraction by adding CBAM after each of the five blocks of the VGGNet model on a large-scale plant disease dataset. In this study, given the use of a small-scale dataset, optimal results were achieved by incorporating CBAM only after Block5 of VGGNet and Block4 of ResNet. The structure of our proposed model is detailed in Figure 17.

### 4.4. Platform and Hyperparameters

Even with the use of transfer learning, there are instances where the pre-trained model may not perfectly adapt to the specifics of a given task or new dataset. Therefore, it becomes necessary to experiment with various hyperparameters to tailor and optimize the model for the specific task. In this study, we compared different optimizers and regularization techniques using the same dataset to find the optimal hyperparameters. We looked at two types of optimizers: Momentum and Adam. For regularization techniques, we evaluated L1 Lasso and L2 Ridge. Additionally, we experimented with various settings for learning rate, weight decay, dropout, and batch size to optimize our results.

In this research, we utilized PyCharm Community Edition 2023.2.1 for building and generating deep learning models; Anaconda3 for managing library files; and LabelImg v1.5.2 for annotating. The details regarding the experimental platform and recommended hyperparameters are presented in Table 8.

### 4.5. Model Evaluation

In this study, we tested 10 models using a 5-fold cross-validation (5-fold CV) method, including VGGNet, ResNet, and these models integrated with CBAM (VGGNet+CBAM and ResNet+CBAM). We recorded the average precision (AP) for the three categories “greening”, “healthy”, and “others”, as well as the mean AP (mAP) across all categories to compare the overall performance of the models.

#### 4.5.1. Evaluation Metric

Given the imbalanced nature of the three labels in our dataset, with a majority being “greening”, there exists a risk that the model could achieve high accuracy by predominantly predicting the majority labels while neglecting the minority labels. To address this potential bias and to evaluate the model’s performance more comprehensively, AP [26] was employed as the primary evaluation metric. By calculating the AP for each category and using their mean value mAP, it is possible to evaluate the overall performance of the model. In this study, we used AP and mAP to evaluate the detection capability of each category and the overall performance of the models.

#### 4.5.2. k-Fold Cross-Validation

k-fold CV [27] is a widely used method for evaluating the performance of models in deep learning. This method involves dividing the dataset into k mutually exclusive folds and alternately conducting training and validation to aim for a more accurate estimation of the model’s performance. Specifically, k cycles of training and validation are carried out, where one of the k folds is selected as the validation dataset in each cycle, and the remaining k − 1 folds are used as the training dataset. Performance evaluations are recorded in each cycle, and the average of these evaluations is calculated to estimate the model’s average performance.

**Figure 18:**
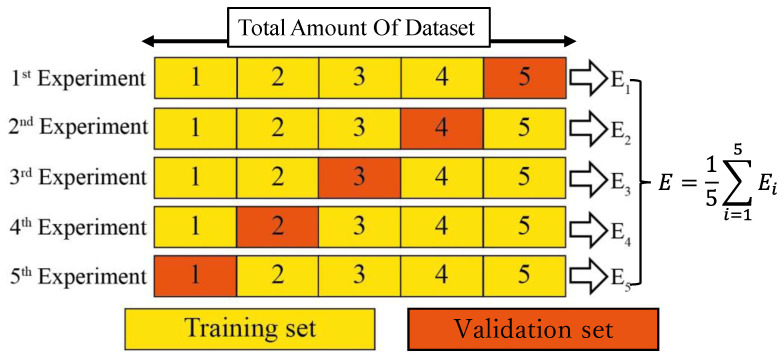
The 5-Fold cross-validation.

Using k-fold CV allows all data to be used for both training and validation, enabling a fairer assessment of the model’s generalization ability, particularly when dealing with small datasets. This maximizes data utilization and evaluates the model across multiple independent validation sets, making the performance estimation more stable and reliable. Typically, k is chosen between 5 to 10, but for our small datasets, k was set to 5. The approach of a 5-fold CV is illustrated in Figure 18.

In detail, we initially divided the 82 collected images randomly into 5 folds, labeled 1 through 5. We then applied data-augmentation techniques of rotation and flipping to the images in each fold, ensuring that both original and augmented images remained within the same fold. For each iteration of the 5-fold CV, 4 folds (e.g., 1, 2, 3, 4) were used as the training set, and the remaining fold (e.g., 5) served as the validation set. This process was repeated such that each fold acted as the validation set once, thereby completing the 5-fold CV cycle.

### 4.6. Web Application

**Figure 19:**
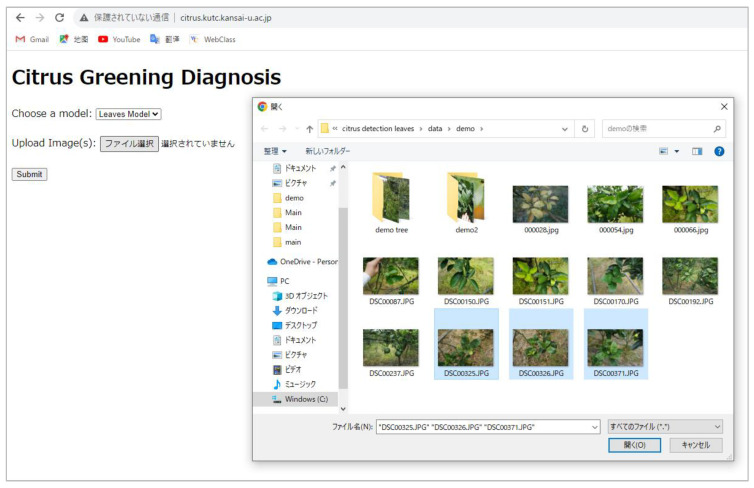
The interface of CG disease-diagnosis web application. Users can click on the button of “Upload Image(s)” option to select the images from the local folder and upload it/them for CG disease diagnosis.

Alongside developing the CG disease-detection model, we also created a web application for practical use. This application, developed using the Django [28] web framework in Python, allows users to upload leaf images (supporting multiple image uploads) for real-time diagnosis. The user interface of our web application is depicted in Figure 19. Local farmers can take images of leaves on branches and upload them to the web application via smartphone or computer. Uploaded images are transferred to a server computer in the laboratory for disease diagnosis. Frames are drawn directly on the targeted leaves, displaying the classification category and confidence scores on each frame. The diagnosis results are then immediately shown as output images on the web application. The web application is hosted on the server of the Faculty of Informatics at Kansai University and is accessible via the following URL: citrus.kutc.kansai-u.ac.jp.

Using the web application allows for the real-time monitoring of disease conditions, enabling the early diagnosis and management of diseases, which can reduce the spread and impact of the diseases. Additionally, by accurately identifying and treating infected trees, the use of chemical pesticides can be reduced, contributing to environmental protection and reducing production costs.

## 5. Discussion

This study focuses on identifying optimal networks and solutions for the simple and efficient detection of CG disease in field applications. We explored the Faster R-CNN architecture with transfer learning, which demonstrated strong recognition capabilities even under challenging conditions, such as distant targets or backgrounds lacking similar objects, highlighting its robust anti-interference abilities. Users can take advantage of our CG disease-diagnosis system by uploading photos directly from the field to our web application for real-time diagnosis, proving highly practical for immediate use. However, transfer-learning models, which are built on limited datasets, typically excel within similar feature spaces but may struggle with out-of-domain data [29]. 

Our data collection was restricted to leaves from a single citrus variety, gathered only under sunny conditions in January, and from trees aged 5 to 7 years. These limitations could impact the effectiveness of the model, for instance, when applied to different citrus varieties. Nevertheless, CG disease exhibits minimal variation in disease characteristics (appearance and manifestation) across different seasons and varieties [30]. The universality of these disease characteristics suggests that our system could be effectively adapted for use with other types and in various regions. Future improvements will focus on enhancing the model’s versatility. We plan to expand our dataset to include a wider range of citrus species, age groups, and lighting conditions, aiming to address variations in leaf color and size that could affect recognition accuracy. Additionally, by collecting and training samples from other plants using our proposed method, we believe this will also help in diagnosing other plant diseases.

To enhance the model’s ability to recognize CG disease, we presented a novel approach by integrating the CBAM with VGGNet and ResNet models, marking the first attempt within the field to conduct a precision comparison using this combination. Table 9 shows the performance difference between models before and after the integration of CBAM with 5-fold CV. Models with positive values were improved by the integration of CBAM and vice versa.

Table 9 demonstrates that the integration of CBAM yielded a notable improvement in the AP for CG disease detection, with enhancements ranging from 1.06% to 2.02%. This underscores the effective role of CBAM in enhancing feature extraction specific to CG disease. For the ResNet50 model, there was an increase of 1.71% in detecting CG disease; however, this was accompanied by declines of 0.45% and 0.35% in the “healthy” and “others” categories, respectively. This suggests that due to the relatively shallow architecture of ResNet50, the addition of CBAM may lead to an over-reliance on attention-weighted features, potentially resulting in the neglect of other pertinent information or an inability to fully capitalize on the sophisticated features offered by CBAM, thus impacting the accuracy of identification. However, in terms of overall model performance, VGG16 and VGG19 exhibited enhancements of 1.81% and 2.01%, respectively, while ResNet50, ResNet101, and ResNet152 achieved improvements of 0.30%, 1.15%, and 1.58%, respectively. This indicates a trend that increasing the model depth correlates with greater overall performance improvements. This suggests that for small-scale target-detection tasks requiring the learning of a great number of detailed features, more complex models with sufficient capacity to learn and utilize these enhanced features may benefit more substantially from the integration of CBAM.

Although we successfully enhanced the feature extraction for CG disease by combining the CBAM with VGGNet and ResNet models, the highest AP achieved was 89.92% with the ResNet152+CBAM model. To further improve the detection capability for CG disease, it is worth exploring the potential for increased detection accuracy through the integration of other CNN models like EfficientNet [31] or ViT [32] with other attention mechanisms such as ECA-Net [33]. Moreover, to further improve the practical efficiency of our system, we aim to develop it to be capable of extracting frames from videos taken with smartphones or drones to diagnose diseases. Essential improvements in the response speed of the web application, such as using other object-detection architectures like RetinaNet [34] or YOLOv7 [35], which have a faster object-detection speed, are also necessary. Furthermore, by registering disease information in a geographic information system, it is possible to track the spread trends of CG disease within a region and provide a scientific basis for disease prediction and prevention. However, the accuracy of location information at the level of individual trees is insufficient with smartphone Global Positioning System (GPS) functions, so management is currently performed at the plantation level. In practice, in the mountainous areas of Chiang Mai Province, Thailand, the HRDI is developing a geographic information system for managing plantations, and it is believed that the results of this research can be utilized there.

## 6. Conclusions

In this study, we explored a fundamental yet innovative approach for diagnosing CG disease using in-field images of citrus leaves taken in orchards in Thailand through transfer learning with the Faster R-CNN architecture. The focus of our research was to compare the effects of transfer learning using VGGNet and ResNet and the integration of the CBAM attention mechanism into CNN models, providing valuable insights for future research. We used AP and mAP as the evaluation metric and conducted a 5-fold CV, assessing a total of 10 models based on VGGNet and ResNet. The key findings are as follows:

- The ResNet models demonstrated superior performance compared to the VGGNet models;
- The integration of CBAM into VGGNet and ResNet models yielded outstanding improvement;
- The ResNet152+CBAM model performed best in both the accuracy of CG disease detection and overall performance;
- The implementation of Faster R-CNN with in-field images notably improved the efficiency and practical application of CG disease detection.

By using our system for real-time CG disease diagnosis, the efficiency of early in-field detection will be improved with a relatively high level of accuracy. Given the severe impact of CG disease on global citrus production, the results of this study facilitate the development of techniques to mitigate this disease problem and even support economic citriculture to some extent. Furthermore, this study not only contributes to the stable production of citrus and the improvement of plant quarantine systems but also has the potential to be applied to research on other plant disease diagnoses.

## Figures and Tables

**Table 1:** The 5-fold CV results of VGGNet and ResNet.

Model/Label	Greening	Healthy	Others	mAP
VGG16	81.44	90.63	94.23	88.77
VGG19	79.04	89.04	90.29	86.12
ResNet50	83.49	93.20	97.18	91.29
ResNet101	85.07	91.54	96.88	91.16
ResNet152	84.51	92.24	95.48	90.74

**Table 2:** AP of “greening” with VGGNet and ResNet in each experiment.

Model/Experiment	AP of “Greening” (%)				
	(1/5)	(2/5)	(3/5)	(4/5)	(5/5)
VGG16	83.26	82.63	86.20	79.47	75.62
VGG19	79.96	79.46	84.54	78.38	72.84
ResNet50	86.32	83.21	86.62	83.20	78.09
ResNet101	87.38	85.36	87.96	84.23	80.40
ResNet152	86.94	84.77	87.59	84.90	78.34

**Table 3:** mAP of VGGNet and ResNet in each experiment.

Model/Experiment	mAP (%)				
	(1/5)	(2/5)	(3/5)	(4/5)	(5/5)
VGG16	89.34	90.35	91.54	86.83	85.77
VGG19	85.24	88.51	88.08	84.71	84.06
ResNet50	91.95	92.03	91.60	90.41	90.46
ResNet101	91.92	93.25	91.09	90.82	88.75
ResNet152	90.94	92.77	90.87	91.40	87.72

**Table 4:** The 5-fold CV results of VGGNet+CBAM and ResNet+CBAM.

Model/Label	Greening	Healthy	Others	mAP
VGG16+CBAM	83.45	92.56	95.71	90.57
VGG19+CBAM	80.76	90.85	92.77	88.13
ResNet50+CBAM	85.20	92.75	96.83	91.59
ResNet101+CBAM	86.13	93.14	97.68	92.32
ResNet152+CBAM	86.52	92.56	97.90	92.33

**Table 5:** AP of “greening” with VGGNet+CBAM and ResNet+CBAM in each experiment.

Model/Experiment	AP of “Greening” (%)				
	(1/5)	(2/5)	(3/5)	(4/5)	(5/5)
VGG16+CBAM	86.42	83.99	87.17	81.88	77.78
VGG19+CBAM	81.55	81.54	85.25	80.43	75.04
ResNet50+CBAM	87.95	85.36	87.98	84.72	79.99
ResNet101+CBAM	88.68	86.37	89.00	85.14	81.44
ResNet152+CBAM	89.00	86.48	89.92	85.76	81.46

**Table 6:** mAP of VGGNet+CBAM and ResNet+CBAM in each experiment.

Model/Experiment	mAP (%)				
	(1/5)	(2/5)	(3/5)	(4/5)	(5/5)
VGG16+CBAM	90.86	92.00	91.96	89.25	88.80
VGG19+CBAM	88.43	89.64	88.86	88.74	84.97
ResNet50+CBAM	92.29	92.91	91.49	91.16	90.12
ResNet101+CBAM	93.06	93.78	93.10	91.38	90.27
ResNet152+CBAM	92.17	94.02	93.31	91.27	90.86

**Table 7:** The number of annotations and images with our dataset.

Label	Greening	Healthy	Others *
Number of annotations	2544	998	582
Number of images	656		

* Diseases other than CG disease.

**Table 8:** Platform and hyperparameters used in experiments.

Platform		Hyperparameter	
Operating system	Windows 11 Pro	Learning rate	0.001
CPU	i9-12900 K	Weight decay	0.001
GPU	NVIDIA RTX 4070, 12 GB	Regularization	L2 ridge regression
RAM	64 GB	Optimizers	Adam
Python	3.7	Dropout	0.5
Tensorflow	2.10.0, gpu	Batch size	256
CUDA	11.4	RPN batch size	256
cuDNN	8.9.5	Max iteration	40,000

**Table 9:** Performance difference before and after the integration of CBAM.

Model/Label	Greening	HealthY	others	mAP
VGG16	2.01	1.93	1.48	1.81
VGG19	1.73	1.82	2.48	2.01
ResNet50	1.71	−0.45	−0.35	0.30
ResNet101	1.06	1.6	0.80	1.15
ResNet152	2.02	0.32	2.42	1.58

## Data Availability

The data presented in this study are available upon request from the corresponding author.

## Abbreviations

5-fold CV (5-fold cross-validation); AP (Average Precision); CAM (Channel Attention Module); CBAM (Convolutional Block Attention Module); CG (Citrus Greening); CNN (convolutional neural network); ECA-Net (Efficient Channel Attention–Neural Network); GPS (Global Positioning System); HRDI (Highland Research and Development Institute); ILSVRC (ImageNet Large-Scale Visual Recognition Challenge); mAP (mean average precision); PCR (Polymerase Chain Reaction); R-CNN (Region-Based Convolutional Neural Networks); ResNet (Residual Neural Network); SAM (Spatial Attention Module); VGGNet (Visual Geometry Group Neural Network); YOLO (You Only Look Once).
